# Aristotle: stratified causal discovery for omics data

**DOI:** 10.1186/s12859-021-04521-w

**Published:** 2022-01-15

**Authors:** Mehrdad Mansouri, Sahand Khakabimamaghani, Leonid Chindelevitch, Martin Ester

**Affiliations:** School of Computing Science, Simon Fraser University, 8888 University Drive, Burnaby, CA USA

**Keywords:** Causal discovery, Stratification, Biclustering, Quasi-experiment

## Abstract

**Background:**

There has been a simultaneous increase in demand and accessibility across genomics, transcriptomics, proteomics and metabolomics data, known as omics data. This has encouraged widespread application of omics data in life sciences, from personalized medicine to the discovery of underlying pathophysiology of diseases. Causal analysis of omics data may provide important insight into the underlying biological mechanisms. Existing causal analysis methods yield promising results when identifying potential general causes of an observed outcome based on omics data. However, they may fail to discover the causes specific to a particular stratum of individuals and missing from others.

**Methods:**

To fill this gap, we introduce the problem of stratified causal discovery and propose a method, Aristotle, for solving it. Aristotle addresses the two challenges intrinsic to omics data: high dimensionality and hidden stratification. It employs existing biological knowledge and a state-of-the-art patient stratification method to tackle the above challenges and applies a quasi-experimental design method to each stratum to find stratum-specific potential causes.

**Results:**

Evaluation based on synthetic data shows better performance for Aristotle in discovering true causes under different conditions compared to existing causal discovery methods. Experiments on a real dataset on Anthracycline Cardiotoxicity indicate that Aristotle’s predictions are consistent with the existing literature. Moreover, Aristotle makes additional predictions that suggest further investigations.

**Supplementary Information:**

The online version contains supplementary material available at 10.1186/s12859-021-04521-w.

## Background

The goal of causal discovery is to infer the cause-effect relationships between an outcome variable and a number of explanatory variables, called features, which pinpoint the mechanism through which changes in the features result in changes in the outcome [1]. Causal discovery is used in various fields to discover the causes of complex outcomes, from economics to medicine [2–5]. In the life sciences, genomics, transcriptomics, proteomics and metabolomics data, often referred to as omics data, have experienced an explosive growth rate over the past decade, making them great candidates for causal discovery [6].

For instance, anthracycline cardiotoxicity is a multi-factorial Adverse Drug Reaction (ADR), whose main mechanisms are believed to be the inhibition of Topoisomerase 2$\beta$ and the reaction of reactive oxygen species [7]. This means that the *rs*2229774 mutation in the *RARG* gene, which influences Topoisomerase $2\beta$, would cause toxicity in some, but not all of the anthracycline patients [8]. Current causal analysis methods may lack the statistical power for detecting rs2229774 mutation as a cause for the ADR when looking at the whole population. This causal variant would be dismissed by a current causal analysis methods on the whole population. This is because rs2229774 is absent in those cases where the ADR is caused by the reactive oxygen species mechanism, as there would be patients without this variant in the *RARG* in which anthracycline cardiotoxicity would nevertheless occur.

Randomized controlled trials, where participants are randomly assigned to a case group that undergoes a treatment and a control group that does not, are considered the state-of-the-art approach for testing causality [9]. However, due to costs and ethical restrictions, randomized controlled trials are impractical to conduct in many scenarios.

The increasing availability of observational data has motivated the development of data mining methods for causal discovery from observational data in the past three decades, and the structure learning approach has been the center of these developments [10]. The goal of structure learning is to produce a graph whose edges represent causal relationships between the variable nodes [11]. The common constraint-based structure learning methods achieve this by employing a series of statistical tests to evaluate the conditional independences between the variables to incrementally construct the graphical structure.

One of the major developments in structure learning is the PC-algorithm [1], which is designed to efficiently discover the causal structure in the absence of unobserved confounders. FCI [12], RFCI [13] and their subsequent modifications [14–16] were able to find causal relations in the presence of unobserved confounders by performing additional conditional dependency tests, to improve the speed of the algorithm by compromising on the dependency assumptions, and to eliminate the dependency on the order of tests by preparing the model for possible orientation rule effects. Another direction of research has been to investigate local causal discovery methods [17], which focus on the Markov blanket to find only those features that are the direct causes of the outcome. However, the computational complexity of structure learning remains exponential in the number of variables [17], which make it infeasible for omics datasets with hundreds of thousands of variables [18].

In recent years, a new direction of research in the data mining community for causal discovery from observational data has emerged. The new methods consist of a filtering algorithm for discovering candidate features from the set of all features, coupled with a Quasi-Experimental Design (QED) or a similar statistical hypothesis testing process for evaluating the validity of those candidate causes. This paradigm for causal discovery promises to scale to larger datasets because the filtering algorithm drastically reduces the number of hypotheses to be tested by QED.

In [19], candidate features are selected through an association rule mining algorithm and tested in a series of retrospective cohort studies to evaluate the causality of the candidates. In HUME [20], a network of co-occurrence between features and outcomes was used to determine the most likely candidate feature-outcome combinations and their potential confounders, and the causality of the candidates was evaluated by a matched pairs QED.

**Fig. 1:**
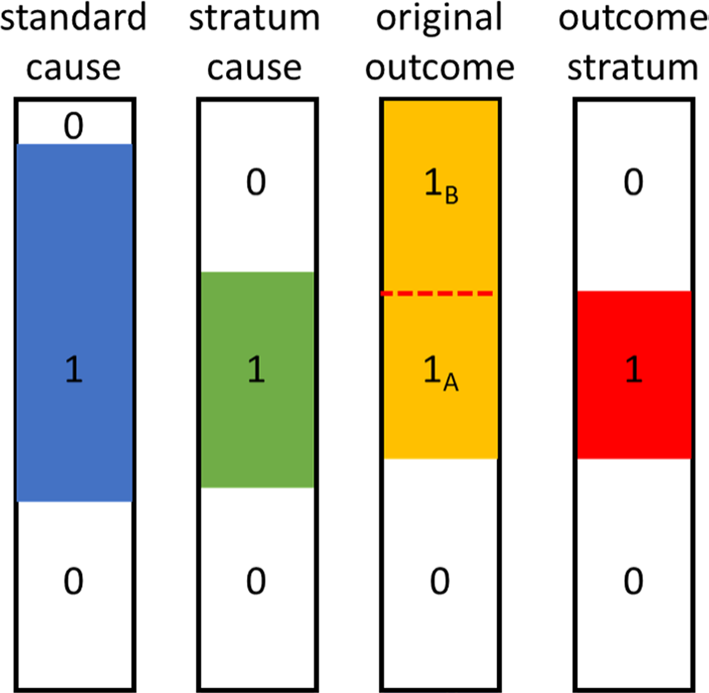
Diagram representing four variables (columns) across different sample (rows). A standard cause (blue) is highly associated with the original outcome (yellow), and can be identified by most measures of association. However, a stratum cause (green) that only affects the outcome in a particular type of samples ($1_A$), could be missed from simple association with the outcome, and requires comparison against its corresponding outcome stratum (red) to be identified confidently

All of the aforementioned methods have the tendency to discover the causes that would try to explain all positive cases in the outcome. However, this gives low significance to causes that occur only in a particular subpopulation of the positive cases (Fig. 1).

To perform causal discovery in such scenarios, this paper introduces the problem of stratified causal discovery (SCD), the simultaneous discovery of sub-populations and their corresponding causal mechanisms. We show that if the outcome is modified to represent the outcome strata instead of the original outcome, the less prevalent causal mechanisms are more likely to be captured. Here, an outcome stratum is a subset of cases with a positive outcome that share a causal mechanism.

We present Aristotle, the first method for solving SCD. Aristotle is a multi-phase algorithm that tackles the above challenges by using a novel divide-and-conquer scheme that utilizes biclustering for finding the promising strata and candidate causes and QED to identify the stratum-specific causes. Aristotle is capable of analyzing high dimensional omics data and allows the incorporation of prior domain knowledge about potential confounders and the grouping of biological features.

Our extensive experiments on synthetic and real datasets demonstrate that Aristotle effectively discovers potentially causal features and strata and clearly outperforms the compared state-of-the-art methods.

Rest of the paper is organized as follows. In “Problem definition” section we formally introduce the stratified causal discovery problem and describe the inputs, the outputs, and the assumptions. In “Methods” section we provide an overview of Aristotle, a justification for its design, and a detailed explanation of each of its phases. Finally, in “Results” section we present our experiments on synthetic and real data and discuss our findings.

## Problem definition

The goal of the SCD problem is to identify the causal features involved in different mechanisms associated with an outcome, such as an ADR. The outcome is assumed to have one of two possible values: positive (e.g. an ADR observed) and negative (e.g. no ADR observed). We are interested in the mechanisms corresponding to the positive outcome. Each distinct mechanism is captured by a different hidden stratum of samples, and for every stratum, we want to find the features that potentially cause the positive outcome. Hence, SCD is defined as the problem of identifying strata of samples with positive outcome such that for each stratum, there exist a group of features at statistically significant association with membership in the stratum, after controlling the effect of confounders. Here, we follow the strict notion of causality, which expects that the outcome is positive almost always when and only when the feature group is positive. There are two types of confounders that need to be controlled to avoid endogeneity: (1) natural confounders which are the variables that are known to have association with the outcome, and may include a wide variety of variables such as demographic and clinical attributes, and (2) omics confounders which are features that have a statistically significant association with the outcome. Formally, the inputs are as follows:

- An outcome vector $R \in \{-1,+1\}^{n}$, where $r_i=+1$ if sample *i* has the positive outcome and $r_i=-1$ otherwise.
- A matrix of omics profiles $G \in \{0,1\}^{p \times n}$, where $g_{.i}=\{g_{1i},...,g_{pi}\}$ indicates the feature values for sample *i*. If sample *i* has feature *j* present, this is denoted by $g_{ji}=1$, and $g_{ji}=0$ otherwise. *G* can contain a large collection of omics data such as mutations, gene expression, and copy number variations [21].
- A matrix of confounder profiles $Z \in \{0,1\}^{m \times n}$, where $z_{.i}=\{z_{1i},...,z_{mi}\}$ indicates the state of the confounders for sample *i*.
- Prior knowledge about the relationships between the features (e.g., pathways as functional groups of genomic features). As described later in “Group Features Based on Background Knowledge” section, this knowledge is used to group related features to resolve the high-dimensionality issue by dividing the large problem into smaller pieces. This input is only needed for high-dimensional inputs with hundreds of thousands features.

The outputs are:

- A (disjoint) grouping $\{P_1,...,P_K\}$ of the sample population *P* such that $\cup _k P_k = P$. Equivalently, this is the assignment of each sample to one of *K* strata. Each stratum contains a subset of the population with similar outcome (for almost all *k* and all $a,b \in P_k$, $r_a=r_b$).
- For each stratum with positive outcome, a set of features that are potentially causing the outcome in the stratum. A causal relationship of the feature *j* to the outcome *r* for strata $P_k$ given confounders *Z* means that, after correcting for the effect of *Z*, there is significant evidence suggesting that for the samples in $P_k$, the value of the feature *j* is responsible for determining the outcome *r*.

Values of features and outcome are assumed to be discrete for three reasons: (1) discrete data can be modeled through Multinomial distribution, which reduces the complexity in training and evaluation of the models, (2) discrete omics data have been shown to improve the prediction accuracy and generality of the trained model [22, 23], (3) most of the currently available high dimensional omics data are discrete in nature [24]. For effective discretization of continuous data (gene expression for example) for the strata analysis, we suggest the method used in [25].

**Fig. 2:**
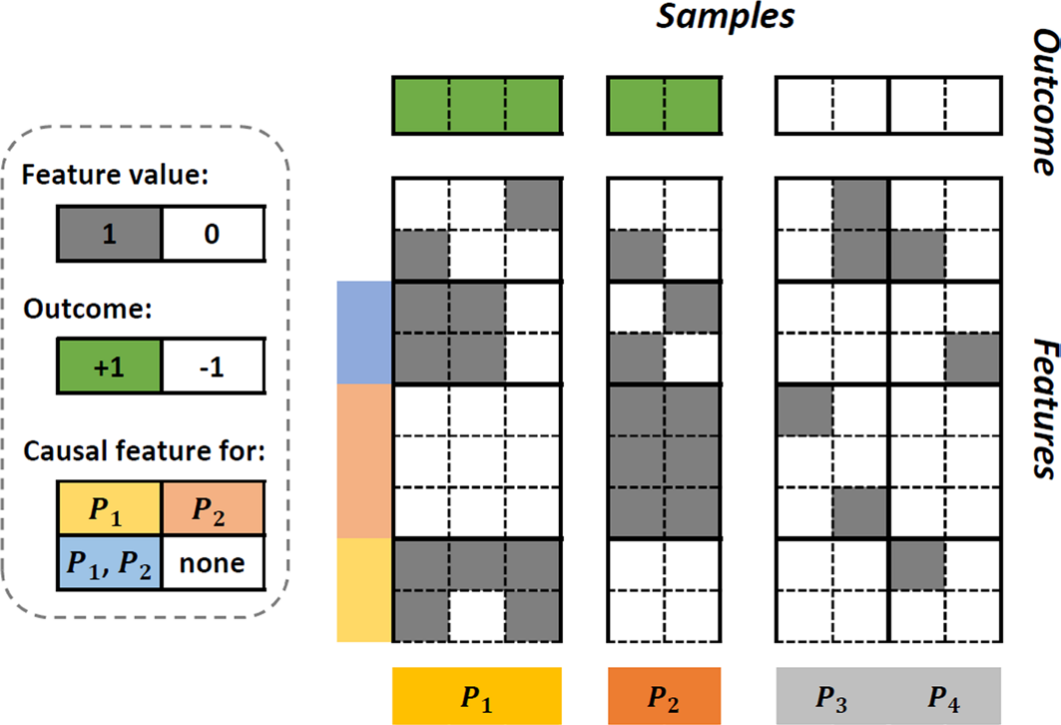
A sample dataset illustrating stratum-specific and general causal features

It should also be noted that unlike the classical causal discovery methods where the causes are considered based on their adjusted association with the positive outcome, in SCD the causes are considered based only on the outcomes corresponding to the particular stratum. Figure 2 illustrates these two types of causes.

There are two main challenges in solving the SCD problem. The first challenge is the small number of samples combined with a very large number of features, which is typical of most omics datasets. This problem is exacerbated in SCD because stratification (1) further thins out the number of samples for each hypothesis, and (2) multiplies the number of possible causes by the number of strata. This in turn increases the amount of penalty of the multiple hypothesis testing of the causal analysis and makes it less likely to find the right cause, especially if the number of samples is small. The second challenge is to identify the hidden strata that reflect the underlying causal mechanisms; these are not known a priori, and, if poorly chosen, may negatively impact the validity of the results.

## Methods

The overall design of ARISTOTLE is based on a novel divide-and-conquer scheme that breaks the set of features into groups and aggregates the significant sample and feature patterns. To do this, ARISTOTLE utilizes supervised biclustering to identifying the promising strata and features, and matching QED to evaluate the causality of features with respect to an identified stratum.

ARISTOTLE’s solution to SCD’s first challenge is to select a shortlist of candidate causes from the set of features, based on their association with the effect, which is the causal strata in SCD. However, the true strata are unknown, which brings us to SCD’s second challenge of simultaneously stratify the samples and identify the candidate causes. This can be solved using a biclustering that jointly groups features and samples based on their association, which could also be used to score and filter the features.

However, due to the high dimensionality, it is infeasible to perform the computationally complex biclustering on all features simultaneously. Hence, for high-dimensional omics data, ARISTOTLE has to divide the features into groups, perform the biclustering to find the candidate features and strata locally, i.e. separately within each group, and then merge the results to form a data matrix with fewer but more relevant features. After the features are reduced to candidate features, biclustering can be reused, this time addressing the second challenge. Grouping of the features can be performed randomly, if no prior grouping knowledge is available, or skipped if the problem is not very high-dimensional, in which case all features are set in one group. Finally, Aristotle needs to evaluate the causality of the association between each of the candidate features and each of the positive strata. QED is used for causal inference which is one of the best approaches for causal inference from observational data of small sample size created by the first challenge.

**Fig. 3:**
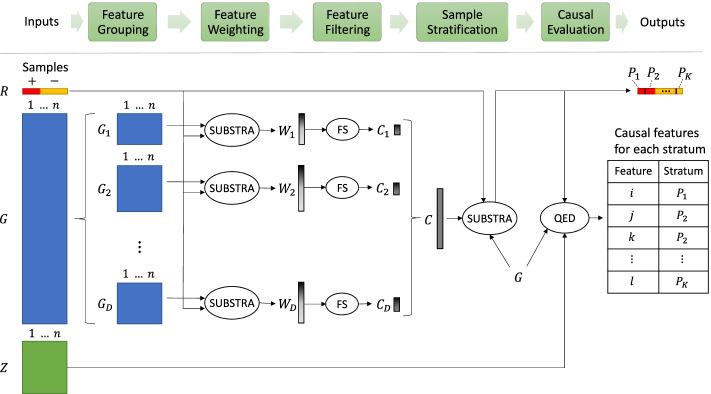
Overview of Aristotle. The data are shown by rectangular blocks and the methods are shown by ovals. *P*, *R*, *G*, and *Z* are defined in “Problem definition” section $G_1$ to $G_D$ are the subsets of *G* corresponding to feature groups produced in the Feature Grouping phase (see “Group Features Based on Background Knowledge” section). $W_1$ to $W_D$ are the feature weights produced by biclustering for each feature group. $C_1$ to $C_D$ are the sets of the top weighted features in each of the feature groups that the feature selection method (indicated by FS in the diagram) chooses from groups $G_1$ to $G_D$ given the weights $W_1$ to $W_D$, respectively.$P_1$ to $P_K$ are the sample strata produced by biclustering. Quasi-experimental design (indicated by QED in the diagram) evaluates the candidate features in the set $C=\cup _{d=1}^{D} C_d$ for causality with respect to their corresponding sample strata and confounders *Z*. The outputs of QED are tuples that indicate the causal pairs of feature and stratum

In short, Aristotle consists of the following five steps, which are illustrated in Fig. 3:

1. Group features of the input data into *D* groups $G_d$, $1 \le d \le D$, based on background knowledge;
2. Assign weights $W_d$ to the features of each group $G_d$;
3. Filter the set of features of each group $G_d$ given their corresponding weights $W_d$, resulting in a set of locally selected features $C_d$;
4. Stratify the samples based on the set of candidate features $C=\cup _{d=1}^D C_d$ and the outcome *R*, resulting in *K* strata $P_k$, $1 \le k \le K$;
5. Evaluate the causality of the candidate features *C* with regard to the outcome in each stratum $P_k$.

Further details of each step are provided in the following sections.

### Group features based on background knowledge

This phase groups features into *D* possibly overlapping subsets $G_d$ ($1 \le d \le D$), such that $\cup _{d=1}^D G_d \subseteq G$. Each group should be small enough to be handled efficiently by biclustering, and at the same time, there should be a meaningful relationship between the features that are in the same group, so that the results of the downstream analysis are relevant.

Because biological pathways are the functional units inside the cell, their use to produce distinct views from omics data is commonplace [26, 27]. Therefore, in our experiments, we choose to form feature groups that correspond to Kyoto Encyclopedia of Genes and Genomes (KEGG) pathways [28] to reflect the real-world implications of the functional effects of pathways. The features that did not correspond to any pathway are discarded.

### Assign weights to features

In this phase, for each feature group, Aristotle needs to bicluster features and estimate strata-based feature weights. For this task ARISTOTLE uses SUBSTRA [25], a state-of-the-art probabilistic supervised biclustering method. SUBSTRA takes the omics profiles of a set of samples along with the outcomes, and produces three inter-related outputs: (1) sample strata, (2) feature clusters, and (3) feature weights. SUBSTRA learns these outputs through an iterative approach that simultaneously optimizes two objectives: biclustering quality and predictive performance.

We chose SUBSTRA for three reasons. First, SUBSTRA assumes similar outcomes for samples in each stratum. This is important for consistency with the notion of stratum-specific causality in the SCD problem (“Problem definition” section). Second, SUBSTRA learns feature weights according to their relevance to the outcome, and uses these weights when computing the strata. Accordingly, the feature weights, the strata, and the outcome depend on each other in SUBSTRA, so the produced strata are related to the significant features which are involved in different mechanisms of the outcome. Third, the feature weights produced by SUBSTRA indicate the amount of dependency between the features and the outcome, and can be used for filtering out irrelevant features and narrowing down the set of candidate causal features. This feature filtering helps reduce the number of effective hypotheses and avoid the multiple-hypothesis testing penalties, which is the first challenge in SCD. It should be noted that this is only possible because the patterns that cannot reach statistical significance, or are not tested against the outcome, do not need to be taken into account while correcting for multiple hypothesis testing [29, 30]. This assumption is tested in practice in the result section.

### Filter features of each feature group

In this phase, we identify the most promising features based on the weights they received after the Feature Weighting phase. Since SUBSTRA produces weights relative only to the other features in each group, the weights from two different feature groups are not comparable to each other. Accordingly, we apply a Feature Selection algorithm independently to each group. For this task, we use an outlier detection algorithm, which selects features with weights outlying from the distribution of weights in the group.

Specifically, we use the scaled median absolute deviation (MAD) [31] as the boundary above which the weights are considered outliers. There are two reasons for choosing MAD. First, MAD is based on a parsimonious and well-studied equation which requires only one parameter, and adds a minimum level of complexity to the process. Second, MAD is extremely robust to heavy-tail distributions, as we observe for SUBSTRA’s weights. The MAD for the feature weights of a group *d* can be computed using the formula:

1 $$\begin{aligned} MAD_d = \frac{\mathrm {M}\left( |W_d-\mathrm {M} \left( W_d\right) |\right) }{\sqrt{2} \cdot \mathrm {erf^{-1} (1/2)}}, \end{aligned}$$

where $W_d$ is the vector of weights of features for feature group *d*, $M(\cdot )$ is the median of its input argument, and $\mathrm {erf}$ is the Gaussian error function. We can decide whether a weight *w* in group *d* is an outlier, if $w - \mathrm {M}(W_d) > L \times MAD_d$. The parameter *L* behaves similarly to the number of standard deviations, and determines how extreme a weight should be to be considered an outlier. In standard practice, the value of *L* is usually selected as an integer between 2 and 5. With the outlier features from each group in hand, we can aggregate them to form the set of candidate features for causal analysis.

### Sample stratification

To perform the stratified causal analysis, we need to know the sample strata. To compute the sample strata, we only use the selected important features from the Feature Filtering phase, which are expected to be more relevant to the outcome than the other features. Ideally, we want the strata to be related to the outcome by capturing different patterns that define the subsets of the two outcome classes. Therefore, once again, we use SUBSTRA for this task, which forms strata with homogeneous outcomes, such that the patterns of up-weighted features are different between the strata as well as the positive and negative outcomes.

### Quasi-experimental design

After the strata and candidate features are identified, our objective is to evaluate whether there is sufficient evidence for a causal relation between the features and the samples of a particular stratum. As input, our QED takes the sample strata $P_1$ to $P_K$ produced in the Sample Stratification phase, the candidate features *C* produced in the Feature Filtering phase, and the confounders *Z*. The output of QED is a list of tuples, where each tuple includes a causal feature and its corresponding stratum, i.e. the stratum that has a positive outcome due (in part) to having this feature. Each feature can be associated to more than one strata, and each stratum can have more than one causal feature. We achieve this goal using matched pairs QED.

Matched pairs design is used for hypotheses in which the potential causes, known as the treatment, has two possible outcomes, as is the case for the candidate features in our problem. In a matched pairs design, the samples are grouped into pairs. In each pair, one sample has received the treatment feature and the other sample has not, but they are as similar as possible with respect to the confounders. Confounders must include both the natural confounders given in input, as well as the other candidates which were statistically significant in the initial Feature Filtering step [20, 32]. In our pairing approach, we assume two types of constraints. First, we make sure that the confounders *Z* have identical values for the two elements of each pair. Second, we use Manhattan distance of the other candidate features from *C* to form pairs with elements that are as similar as possible to each other. For efficient pairing, Aristotle employs the Hungarian matching algorithm [33] based on the Manhattan distance.

In the next step we use McNemar’s test to check whether the value of the outcome within the pairs is statistically associated with the value of the treatment feature. With this procedure, a large number of hypotheses would be tested for each SCD problem. This increases the chance of some of these hypotheses being incorrectly accepted by chance, which is known as data dredging. To avoid data dredging, a multiple hypothesis testing procedure needs to be used to adjust the significance level [34]. A standard approach for dealing with multiple hypothesis testing is using the False Discovery Rate (FDR). The FDR is defined as the expected proportion of significant findings that are false positives. Like most methods for controlling the FDR, Aristotle requires the number of true null hypotheses [35]. In Aristotle, due to elimination of some of the potential hypothesis during the Feature Filtering, the equivalent number of hypothesis is hard to identify and lies in the wide range from the number of candidates to the total number of features. Therefore, a method for estimating the number of true null hypotheses is used in Aristotle. A well-known procedure for the estimation of the number of true null hypotheses is the adaptive Benjamini-Hochberg [36]. Adaptive Benjamini-Hochberg works based on the graphical interpretation of the $q-q$ plot of *p*-values, which results in a simple stepwise procedure for estimating the number of true null hypotheses.

In summary, in the QED step: (1) for each candidate, samples are paired based on their differences with respect to the candidate feature, confounders, and other features in *C* using the Hungarian algorithm, (2) the *p*-value of the hypothesis corresponding to our matched pairs design is calculated using McNemar’s test with Yates’ correction, and (3) the *p*-values of candidate features are analyzed using the adaptive Benjamini-Hochberg method and a subset of them are reported as the causal features.

## Results

### Experiments on synthetic data

To properly evaluate the performance of Aristotle under different conditions and with known ground truth, we created synthetic datasets. Our goal was to reproduce the biclustering of SNP annotation score [37–39] and gene expression [40] in different ethnic groups and cell types. To reflect the biological reality and the criteria discussed in the problem definition, the generation of our synthetic data is based on the following assumptions:

- Features form clusters and the members of those clusters have similar values across all samples. The sizes of feature clusters follow a Binomial distribution. Some of the feature clusters are causal, meaning that all of the features in those clusters are causal features. The remaining clusters are non-causal, meaning that none of their members is causal.
- Each feature cluster is present in a subset of samples, i.e. the features of the cluster have a value of 1 for those samples but 0 for the others. Each cluster simulates a haplotype [41], a set of Single Nucleotide Polymorphisms (SNP), i.e. mutations of a single position in the genome, that tend to be inherited together.
- The members of causal feature clusters have value 1 only for a subset of patients with the positive outcome (a stratum corresponding to a causal mechanism). There is a one-to-one relationship between the causal feature clusters and the strata of patients with positive outcome.
- On top of the feature clusters, there is another layer of grouping of features which represents the pathways. These pathways are mixtures of different feature clusters; however, they tend to contain features from a small number of different feature clusters, i.e. they have small entropies. To achieve this, we use a process based on the concept of ”rich gets richer”, i.e. a feature *j* is assigned to a pathway *X* with a probability proportional to the fraction of the current features in *X* that belong to the cluster containing *j*. This idea was introduced in the Barabasi-Albert algorithm [42], originally designed for network simulation.
- Both features and outcome variables contain noise. Noise is added to the features and outcomes by flipping a randomly selected portion of the entries.

The parameters of synthetic data generation are shown in Table 1. All parameters are set to be in the same order of magnitude as our ADR data and the typical values of omics datasets [37, 43, 44], which also matches with the marginal distributions in [45]. We evaluate the effect of the last three parameters in this table, which we expected to have the biggest impact on Aristotle’s performance. The effect of varying each parameter is investigated by fixing the other two at their default values shown in Table 1. It should be noted that similar omics data-generation processes in the literature could not be used [45], because they could not produce the biclustering joint distribution reflected in the annotation scores [37].

### Baseline methods

Two other baseline methods are included for benchmarking purposes. First, we use the Really Fast Causal Inference (RFCI) algorithm [13] to compare the accuracy of Aristotle in causal discovery. This method is designed for learning causal relationships between random variables. It infers conditional independence between all pairs of variables assuming arbitrary number of latent (i.e. unmeasured) and selection (i.e. unmeasured variables determining the inclusion of the effect of measured variables) variables, which makes the detection of causal relationships difficult. RFCI improves the computational complexity of FCI [1] by reducing the number of conditional independence tests and conditioning on a smaller number of variables. However, RFCI is still computationally intensive. Therefore, we employed the same divide and conquer approach as in Aristotle. More precisely, RFCI is applied to each pathway separately and then applied again to the union of causal features selected for different pathways by RFCI. This produces the final set of features predicted to be causal for the outcome variable.

Second, to compare Aristotle’s stratification quality, a supervised fuzzy clustering method introduced in [46] is used as benchmark. Similar to SUBSTRA, this method incorporates supervision into the clustering and produces classification scores for features. Their algorithm uses the class label of each point to identify the optimal set of clusters that describe the data, and the obtained clusters are then used to build a fuzzy classifier based on relevant features identified using Fisher-interclass separability method [47]. We used all of the features as input for this algorithm as one would naturally do (i.e. no divide and conquer approach).

**Table 1:** Parameters of synthetic data generation and their values

Parameter	Value
Number of features	100,000
Number of feature clusters	2500
Number of pathways	100
Feature noise	0.05
Fraction of positive samples	0.2
Number of causal feature clusters	2, 3, 4, 5
Outcome noise	0, 0.05, 0.1, 0.2
Number of positive samples	75, 100, 125, 150

The default values are underlined

### Results of experiments

**Fig. 4:**
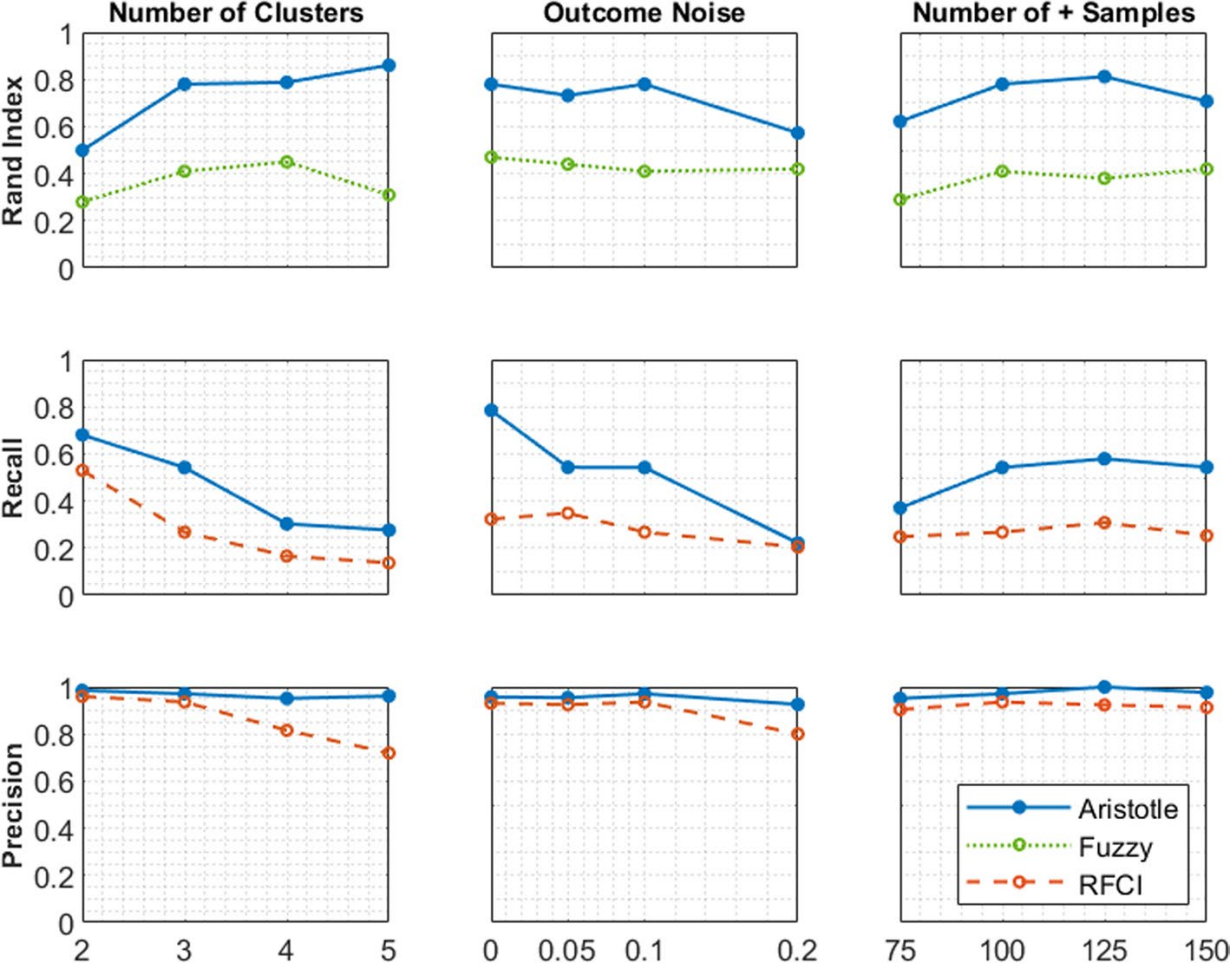
Results for the experiments with the synthetic data

The results are shown in Fig. 4. First, we test whether the strata found by Aristotle match the true strata of the data. We measure this using the Rand index [48], a well-known method for measuring the performance of a clustering algorithm based on an external gold standard. These results show that the Rand index of Aristotle’s strata with respect to the true strata is consistently above 0.5 and reaches a value of about 0.8 in most cases. As expected, the Rand index decreases with increasing noise, but does so slowly. Somewhat unexpectedly, Aristotle tends to be more successful in scenarios with a larger number of strata. This is due to the fact that Aristotle tends to decompose the true strata into subsets due to the effect of non-causal features, resulting in smaller Rand index for cases with fewer strata. The Rand index varies little with respect to the number of samples. Compared to the supervised fuzzy clustering method, Aristotle achieves a consistently and substantially larger Rand index in all experimental settings. Even though both methods use supervision, this indicates a better incorporation of the supervision information in Aristotle through feature weighting.

Second, we evaluate how well Aristotle performs in finding the causal features. We observe that the recall of causal features decreases significantly with increasing percentage of noise and with increasing number of causal feature clusters (see Fig. 4). This may be due to the reduction in size of the positive strata, which results in a weaker signal for each causal feature and consequently lower statistical power. For the same reason, increasing the number of positive samples improves the recall. Aristotle consistently outperforms RFCI, which demonstrates the advantages of stratified causal discovery over the classical methods that discover causes in the whole population. The gap between the two methods decreases with increasing outcome noise, and both methods perform equally poorly (recall $\approx 0.2$) for 20% outcome noise. However, the difference between Aristotle and RFCI becomes more pronounced for higher number of positive samples. This is because RFCI substituting the FCI’s possible separation sets with adjacency sets (to speed up the independency checks) compromises the completeness of the causal graph, hinders it from achieving higher accuracies.

As for the sensitivity of Aristotle’s precision to different dataset parameters, according to Fig. 4, its precision remains at a high level, around 95%, and is always better than RFCI in all the experimental settings we tested. This is consistent with the premise of an FDR of 5%. Although RFCI achieves a similar precision for smaller numbers of strata and levels of noise, the advantage of Aristotle increases for the harder problems with more strata and higher noise levels. To conclude, our experimental results on synthetic data show that Aristotle consistently outperforms RFCI for causal feature discovery.

Lastly, we evaluate Aristotle’s success in identifying causal features among the filtered features. To test this, we compare the performance of Aristotle under the default parameters with two alternative QED settings applied to the filtered features: (1) regular QED without considering the strata, i.e. all positive samples as one group, (2) QED using the ground-truth positive strata as the positive outcome. Aristotle achieves a precision of 0.9306 and a recall of 0.6262. The QED with setting (1) has a precision of 0.95 and a recall of 0.29. The greatly reduced recall is due to the alternative QED’s failure to detect the stratum-specific causal features. This is due to the concentration of the occurrence of causal feature in a small subset of positive samples constituting a stratum. When matching in the regular QED, this small difference fails to separate the positive strata and matching positive samples reduces the support for the hypothesis. This further supports our claim that stratification of the samples can find the potential causes which would have been otherwise missed. The reason for the higher precision of the regular QED is that it results in larger *p*-values in general and, therefore, is more conservative. The QED with setting (2) has a precision of 1 and a recall of 0.69. This indicates that Aristotle has a very similar performance to the alternative method based on the ground-truth. We note that recall is more important than precision in the causal discovery setting, because the computational predictions are often later examined experimentally in order to rule out any false discoveries. However, missing an important true causal feature may be more detrimental from a scientific point of view.

### Experiments on real-world data

We used a dataset about Anthracycline Cardiotoxicity, an Adverse Drug Reaction (ADR) to a class of drugs known as Anthracyclines. More than half of childhood cancer treatment protocols include an Anthracycline. However, the usefulness of Anthracyclines is limited by asymptomatic cardiac dysfunction and heart failure [49].

As discussed earlier in the introduction, Anthracycline Cardiotoxicity is now believed to be a multi-factorial ADR with multiple underlying mechanisms including the inhibition of Topoisomerase 2$\beta$ and the action of Reactive oxidation species [7, 50]. Accordingly, discovering the genetic risk markers of pediatric Anthracycline Cardiotoxicity is a SCD problem.

The particular dataset that we used is provided by the Canadian Pharmacogenomics Network for Drug Safety (http://cpnds.ubc.ca/) and consists of the records of 434 childhood cancer patients treated with Anthracyclines, 90 of which show cardiotoxicity [51]. The input consisted of germline SNP profiles. A series of data preparation steps were carried out on the input. We created four binary features for each SNP locus (position on the genome), representing the four possible values that a SNP can take based on paternal and maternal alleles in combination. This resulted in 2.4 million binary features. The set of confounders that could have impacted the outcome of Anthracycline Cardiotoxicity in the patient consisted of age, gender, dosage, cardio-protectant, cardiac irradiation, Vincristine, and Blastine. The age and the dosage were continuous values that we turned into two binary variables based on their quantiles, resulting in a total of 9 counfounders. For the outcome variable (the presence of an ADR), the patient’s descriptive reaction records were reduced to a binary variable based on the Canadian Pharmacogenomics protocols and indicated whether the patient showed an Anthracycline Cardiotoxicity ($+1$) or not ($-1$).

In the Feature Grouping phase, we mapped the SNPs to genes using the tool introduced in [52]. Then, based on the genes’ membership in the 323 pathways, the SNPs were associated with pathways to form the 323 final groups. The SNPs that did not correspond to any gene belonging to one of the pathways were discarded.

We evaluate the results of Aristotle for the ADR dataset from three different standpoints: (1) by a statistical analysis of distribution of the *p*-values of the discoveries, (2) by comparing the overlap between our discoveries and the known causes, which were deduced from independent medical records and the literature, and (3) by providing biological interpretation for corresponding genes and pathways of the discovered SNPs.

**Fig. 5:**
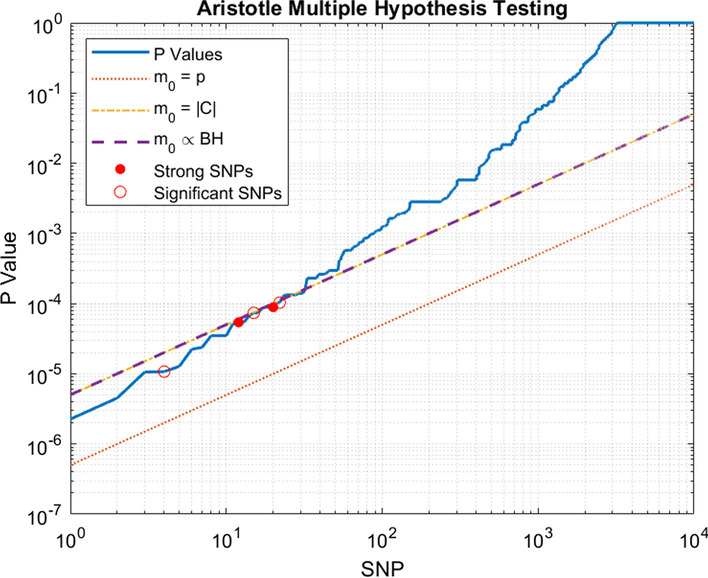
*q*-*q* plot of *p*-values of SNPs. The blue curve is the *p*-values of candidates, calculated by McNemar’s test. The three linear curves show the significance level adjusted by the 0.05 false discovery rate, each based on different assumptions about the number of true null hypotheses. The yellow line assumes that the number of hypotheses is equal to the total number of SNPs in input data, which gives the most conservative possible adjustment. The orange curve assumes that the number of hypotheses is equal to the number of candidates produced by Feature Filtering and used for the statistical test, which gives the least conservative adjustment. The purple line estimates the number of hypothesis according to [36], a method for the estimation of the effective number of hypotheses, which is almost perfectly aligned to the less conservative approach

Figure 5 shows the logarithmic *q*–*q* plot of the distribution of *p*-values of the candidate SNPs computed by Aristotle for the Anthracycline Cardiotoxicity dataset, where SNPs are sorted in ascending order of p-values. The straight lines indicate what would happen under the null hypothesis for different numbers of hypothesis [35]. Interestingly, adaptive estimation of the number of true null hypotheses (the purple line) results in almost the same number of hypothesis as the number of candidate features after filtering (the yellow line), and the corresponding lines align. This indicates that the number of the features passing the filtering of Aristotle is a reasonable estimate of the number of true null hypothesis. The order of magnitude of Aristotle’s *p*-values is similar to those reported in the guideline [53] and HUME [20] and the distribution of the *p*-values significantly deviates away from the straight line, i.e. null distribution). This indicates that our discoveries are comparable to those reported by Hume and Guideline.

The Guideline of Genetic Variants in Anthracycline-induced Cardiotoxicity [53] is used to provide the known causal SNPs for Anthracycline Cardiotoxicity. The guideline reviewed Anthracycline Cardiotoxicity genes reported in the literature using the Canadian Pharmacogenomics dataset as well as independent reproducibility analyses, and categorized those into strong, significant, and notable level of evidence of association. However, this does not mean that the only possibly true relations are guideline relations. We investigate the overlap of the causal SNPs detected by Aristotle with the SNPs discussed in the guideline and HUME [20].

28 SNPs passed the statistical test at the FDR significance level of 5% and were selected by Aristotle as the causal features. The list of the predicted causal SNPs together with their corresponding genes and pathways is provided in Additional file 1: Table S1. Two of the guideline’s three strong SNPs, namely rs2229774 and rs17863783, passed the test with *p*-values of 5.4E-05 and 8.9E-05 respectively. Rs2229774 was also detected by HUME and its validity is carefully studied in [53]. But more importantly, rs17863783 was not detected by HUME. This shows the advantage of Aristotle in finding those factors that are causal for one archetype of cases, but would be missed if all cases are counted the same. Looking more closely, rs17863783 has only a very strong association with Anthracycline Cardiotoxicity for one of the strata, but does not have enough prevalence in the other strata to be detected by existing causal analysis methods.

The guideline’s third strong SNP, rs7853758, was missed due to not being included in any of the pathways. However, if it had been tested under the current QED, it would have resulted in a *p*-value of 0.03, which seems significant in isolation, but would probably not have survived multiple hypothesis testing. However, it should be noted that because the feature groups and consequently the final results are susceptible to change by inclusion of new pathways and significant SNPs, such posterior evaluations are not valid.

Furthermore, three of the guideline’s fourteen significant SNPs passed the test, namely rs17583889, rs10426377 and rs4673, with *p*-values of 1.0E-05, 5.4E-05 and 1.0E-04 respectively. Of the remaining eleven significant SNPs, three had fewer cases than the minimum prevalence threshold, four did not correspond to any of the pathways, two did not pass the filtering process, and the two remaining ones did not have sufficiently low *p*-value to pass the multiple hypothesis testing.

It should be noted that not reporting all of the significant guideline SNPs is not necessarily an undesired outcome. First, as mentioned earlier, significant relations are not the ground truth, and some were even considered insignificant by the guideline and HUME. Second, and more interestingly, there can be associations that are considered significant for the overall population, but lack a sufficient statistical power or association when the hypothesis is focused on specific stratum [54].

Moreover, from a biological standpoint, a significant number of SNPs discovered by Aristotle share the same corresponding genes and pathways. This not only provides evidence for the functional involvement of those genes and pathways in Anthracycline Cardiotoxicity, but also provides further evidence for the validity of Aristotle’s results.

12 of the 28 discovered SNPs share a gene with at least one other SNP. Five of these SNPs, namely rs795887, rs6436364, rs6756107, rs6722420, and rs10755042, are all from the *ACSL3* gene, which is involved in two of the pathways, 1212-Fatty Acid Metabolism and 4146-Peroxisome. Similarly, rs496179 and rs885622 are both from the *DPYD* gene, involved in pathway 410-Beta-Alanine Metabolism. SNPs rs17863783 and rs10426377, which are a strong and a significant SNP from the guideline, respectively, are both from the *SLC28A3* gene in pathway 140-Steroid Hormone Biosynthesis.

The association among SNPs is also present at the pathway level. SNPs rs26848 and rs26849, both in the *PGP* gene, share three pathways with rs545253 in the *MTND4P31* gene. Similarly, SNPs rs16972837 and rs659517, both from gene *RYR3*, and SNP rs607483, are involved in the same pathways 4371-Apelin Signaling Pathway, 4713-Circadian Entrainment and 5010-Alzheimer’s Disease. Moreover, the two *RYR3* SNPs share pathway 4020-Calcium Signaling Pathway with rs11869821. Another example is the SNPS in the *UGT2B7* gene, rs7662632 and rs4356975, which have the same pathways 40-Pentose & Glucuronate Interconversions, 53-Ascorbate & Aldarate Metabolism and 830-Retinol Metabolism, as the guideline’s strong SNP, rs17863783. Interestingly, rs11869821, rs2271235, rs11936348 and rs611954 are each from a different gene, but are from the same pathway. A detailed list of genes and pathways of passing SNPs is available in Additional file 1: Table S1.

## Conclusions

This work introduced the problem of stratified causal discovery and provided a method called Aristotle for solving that problem. Aristotle is applicable for high dimensional datasets, such as omics. It uses background knowledge for decomposing the large feature space into smaller subspaces that are easier to handle. It uses a state-of-the-art stratification method called SUBSTRA for computing feature weights, which are used for feature selection based on outlier detection. Aristotle also detects the hidden strata using SUBSTRA and employs a quasi-experimental design with an adaptive multiple-hypothesis testing for discovering stratum-specific causal features. The main novelty of Aristotle is in its divide and conquer strategy of biclustering and scoring features multiple times in order to ensure that the features and strata used in causal inference are of highest quality.

The method is evaluated using both synthetic and real data. Based on the experiments with synthetic data, in addition to finding the causal features detectable by the conventional causal discovery approach, Aristotle discovered strata-specific causal features. For the case of Anthracycline Cardiotoxicity, Aristotle successfully captured most of the known significant causal features in addition to making new predictions.

A possible limitation of Aristotle is that it consists of five main phases. This might result in the propagation of error from one phase to the consequent phases. Therefore, a direction for future work would be to reduce the number of phases by providing a method that finds the causal features and the corresponding strata in an integrated phase.

## Supplementary Information

**Additional file 1. Table A1:** Predicted causal SNPs for anthracycline cardiotoxicity and their corresponding genes, pathways, and adjusted *p*-values.

## Data Availability

The Anthracycline Cardiotoxicity dataset can be accessed by applying to the CPNDS website (http://cpnds.ubc.ca/), which is a Canada-wide active surveillance network for maintaining a database of over 10,000 patients with ADRs. The source code for Aristotle and synthetic data generator are available at https://github.com/MehrdadMansouri/Aristotle.

## Abbreviations

ADR: Adverse drug reaction
FDR: False discovery rate
KEGG: Kyoto encyclopedia of genes and genomes
MAD: Median absolute deviation
QED: Quasi-experimental design
RFCI: Really fast causal inference
SCD: Stratified causal discovery
SNP: Single nucleotide polymorphisms
