# Supplementary material for: Aristotle: stratified causal discovery for omics data
Source: BMC Bioinformatics. 2022 Jan 15;23:42. doi: 10.1186/s12859-021-04521-w (PMC8760642; doi:10.1186/s12859-021-04521-w)
Supplement: Supplementary file 1 — Additional file 1. Table A1: Predicted causal SNPs for anthracycline cardiotoxicity and their corresponding genes, pathways, and adjusted p-values. [file 12859_2021_4521_MOESM1_ESM.pdf]

Supplementary Material for
Aristotle: Stratified Causal Discovery for Omics Data

Mehrdad Mansouri, Sahand Khakabimamaghani, Leonid Chindelevitch, Martin Ester

A Results on Real-World Data

Table A1: Genes and pathways corresponding to the predicted causal SNPs

Pathway	Gene	SNPs	Adj p-value
		rs795887	2.2312E-05
1212-Fatty Acid Metabolism		rs6436364	3.4963E-05
	ACSL3	rs6756107	5.4212E-05
4146-Peroxisome		rs6722420	1.0325E-04
		rs10755042	1.3302E-04
410-Beta-Alanine Metabolism	DPYD	rs496179	1.2773E-05
		rs885622	1.3302E-04
140-Steroid Hormone Biosynthesis	SULT2B1	rs10426377	1.0740E-05
	UGT1A6	rs17863783	8.8996E-05
4136-Autophagy	BRICD5	rs26848	7.3838E-05
4140-Autophagy		rs26849	1.4048E-04
4150-mTOR Signaling	IGBP1	rs545253	1.3302E-04
4371-Apelin Signaling	RYR3	rs659517	3.4963E-05
4713-Circadian Entrainment		rs16972837	1.0594E-05
5010-Alzheimer's Disease	KCNMA1	rs607483	3.4963E-05
4020-Calcium Signaling Pathway	RYR3	rs16972837	1.0594E-05
		rs659517	3.4963E-05
	PRKCA	rs11869821	4.5205E-06
40-Pentose & Glucuronate Interconversions	UGT2B7	rs7662632	8.8996E-05
53-Ascorbate & Aldarate Metabolism	UGT1A	rs4356975	1.2491E-04
830-Retinol Metabolism		rs17863783	8.8996E-05
4360-Axon Guidance	PRKCA	rs11869821	7.2050E-06
	RASA1	rs2271235	1.3302E-04
	EPHA5	rs11936348	1.4048E-04
	SEMA3A	rs611954	1.5740E-04
4070-Phosphatidylinositol Signaling	PRKCA	rs11869821	4.5205E-06
5231-Choline Metabolism	DGKK	rs17328236	8.6716E-05
4350-TGF-Beta Signaling	LTBP1	rs11124305	1.3302E-04
	SMAD9	rs7986120	1.4048E-04
4514-Cell Adhesion Molecules	RARG	rs2229774	5.4212E-05
	ITGB7	rs2250503	6.7512E-05
4974-Protein Digestion & Absorption	RARG	rs2229774	5.4212E-05
	COL6A5	rs12485916	8.8996E-05
4151-PI3K-Akt Signaling	RARG	rs2229774	5.4212E-05
	PRKCA	rs11869821	4.5205E-06
